# Upscaling of a Batch De-Vulcanization Process for Ground Car Tire Rubber to a Continuous Process in a Twin Screw Extruder

**DOI:** 10.3390/ma9090724

**Published:** 2016-08-24

**Authors:** Sitisaiyidah Saiwari, Johannes W. van Hoek, Wilma K. Dierkes, Louis E.A.M. Reuvekamp, Geert Heideman, Anke Blume, Jacques W.M. Noordermeer

**Affiliations:** 1 Elastomer Technology and Engineering (ETE), Department of Solids, Surfaces and Systems (MS3), University of Twente, Enschede 7522 NB, The Netherlands; sitisaiyidah.s@psu.ac.th (S.S.); j.w.vanhoek@utwente.nl (J.W.v.H.); louis.reuvekamp@apollotyres.com (L.E.A.M.R.); a.blume@utwente.nl (A.B.); j.w.m.noordermeer@utwente.nl (J.W.M.N.); 2 Polymer Engineering, University of Applied Sciences Windesheim, Zwolle 8017 CA, The Netherlands; g.heideman@windesheim.nl

**Keywords:** devulcanization, tire, twin-screw extruder, DPDS, low shear, thermo chemical, Horikx

## Abstract

As a means to decrease the amount of waste tires and to re-use tire rubber for new tires, devulcanization of ground passenger car tires is a promising process. Being an established process for NR and EPDM, earlier work has shown that for ground passenger car tire rubber with a relatively high amount of SBR, a devulcanization process can be formulated, as well. This was proven for a laboratory-scale batch process in an internal mixer, using diphenyl disulfide as the devulcanization aid and powder-sized material. In this paper, the devulcanization process for passenger car tire rubber is upscaled from 15 g per batch and transformed into a continuous process in a co-rotating twin screw extruder with a capacity of 2 kg/h. As SBR is rather sensitive to devulcanization process conditions, such as thermal and mechanical energy input, the screw design was based on a low shear concept. A granulate with particle sizes from 1–3.5 mm was chosen for purity, as well as economic reasons. The devulcanization process conditions were fine-tuned in terms of: devulcanization conditions (time/temperature profile, concentration of devulcanization aid), extruder parameters (screw configuration, screw speed, fill factor) and ancillary equipment (pre-treatment, extrudate handling). The influence of these parameters on the devulcanization efficiency and the quality of the final product will be discussed. The ratio of random to crosslink scission as determined by a Horikx plot was taken for the evaluation of the process and material. A best practice for continuous devulcanization will be given.

## 1. Introduction

Nowadays, a significant part of waste tires is recycled. However, the reuse in cradle-to-cradle loops (tires back into tires) is limited due to the low property profile of the recycled material. The decrease in the quality of the material is partly due to the fact that the rubber already suffered a whole life cycle, but it is also caused by the recycling process itself: the waste rubber is ground and re-plasticized in a process in which not only the crosslinks, but at the same time, also, the polymers are broken down.

Compared to the starting material, the re-plasticized material has a different structure and properties. In order to achieve a quality of recycled rubber that is comparable to virgin material, it needs to be devulcanized, meaning that preferably only the sulfur crosslinks and hardly any of the polymers break down in the recycling process.

Saiwari [1] has shown that after thermo-chemical devulcanization of passenger car tire rubber, using diphenyl disulfide as the devulcanization agent, the devulcanizate has the potential to be reused in tire production. As a follow-up of the batch devulcanization process, as developed by Saiwari, the present report describes the upscaling and transformation into a continuous process. A co-rotating twin screw extruder was chosen for this purpose, because of the good mixing properties and flexibility of configuration. The influences of time, temperature and concentration of devulcanization aid are studied, with the optimal settings for the batch process as the starting point, and the extruder setup and screw configuration are investigated.

Styrene-butadiene rubber (SBR), a major component of ground passenger car tire rubber (GTR), is in particular sensitive to shear, as shown in the work of Saiwari [1]: during the devulcanization process, it easily recombines due to its complex structure, resulting in the formation of inter- and intra-molecular chain fragment recombinations, with a renewed increase in crosslink density as a result [2,3,4,5].

Some preliminary experiments with a high shear setup of a co-rotating twin screw extruder, using the same optimal settings as for the batch process, lead to the conclusion that the material was integrally broken down and the properties of the recycled material were rather poor due to the high shear [1]. Therefore, a low shear configuration of the same type of extruder was chosen as the starting point in this study.

Additional equipment for the devulcanization process had to be developed: a nitrogen purging system, a cooling system for the devulcanizate and swelling equipment for the blend of the particulate rubber with the devulcanization aid and plasticizer. This was finalized during the experiments. On the one hand, this introduced the possibility to study the effect of these systems on the quality of the devulcanizate; on the other hand, it created the situation that trends during the experiments could be compared, but absolute values not.

The main method for analyzing the devulcanization effect is the decrease of the crosslink density determined according to the Flory–Rehner theory, combined with the Horikx presentation of the results [1,6]. Another indication for the quality of the process and material is obtained by stress-strain properties and surface roughness analysis after renewed vulcanization of the devulcanizate. See Table 1 for a comparison between the experimental conditions as found to be optimal for the batch process and those that were used for the continuous process.

## 2. Results and Discussion

### 2.1. Analyses

#### 2.1.1. Decrease in Crosslink Density

**Figure 1:**
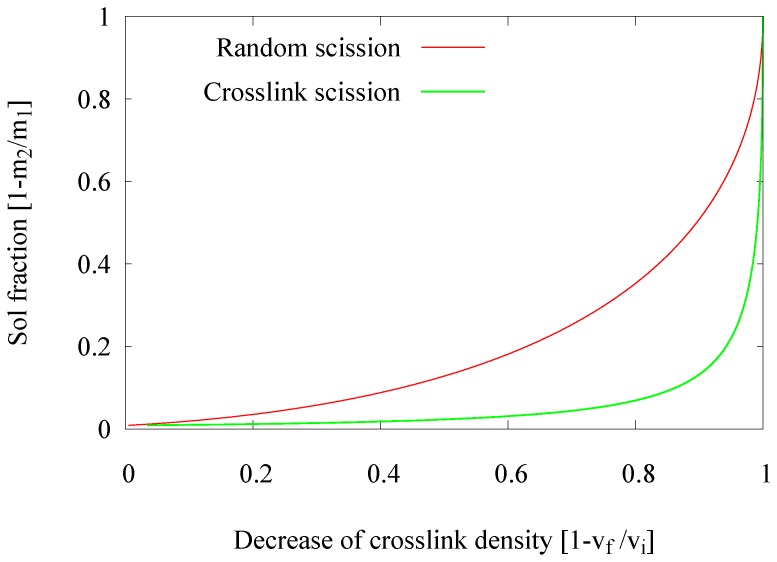
Horikx base plot.

The analysis method based on the crosslink density calculation according to the Flory–Rehner equation and allowing one to estimate the decrease of the crosslink density in a Horikx presentation is extensively described by Verbruggen [6], Verbruggen et al. [7], Saiwari [1], Saiwari et al. [8,9], Charlesby [10] and Horikx [11]: based on the amount of soluble rubber and the amount of solvent absorbed by the rubber, both before and after the devulcanization process, the concentration of crosslinks in the material and thus the decrease of crosslinks during the devulcanization process, can be calculated. Horikx derived a chart in which the decrease of crosslink density is related to the amount of soluble rubber in such a way that both the degree of devulcanization and the quality, in the sense of crosslink versus random chain scission, can be deducted; see Figure 1. With $v_{f}$ the crosslink density after and $v_{i}$ the crosslink density before devulcanization, and $m_{2}$ the sample mass after and $m_{1}$ the sample mass before extraction, the decrease in crosslink density can be presented with $\left(1-\frac{v_{f}}{v_{i}}\right)$ and the sol fraction with $\left(1-\frac{m_{2}}{m_{1}}\right)$. Positions close to the uppermost line indicate random scission of the polymer chains, and positions close to the lower line represent the desired scission of crosslinks only. It should be noted that not more than 90%–95% of a decrease of crosslink density can be achieved with the thermo-chemical devulcanization, as the remaining crosslinks are either stable monosulfidic bonds that cannot be broken by this method or polymer-filler bonds, where the filler is reinforcing carbon black or silica and can therefore not be dissolved.

To determine the amount of soluble rubber, the samples were extracted for two days with boiling acetone followed by three days in boiling tetrahydrofuran and dried at 40 ${\;}^{\circ }C$ in a vacuum oven until a stable weight was measured. The sol content is the amount of soluble rubber, after compensating for the dissolved vulcanization and devulcanization components, processing oils, antioxidants, wax and all other non-rubber additives. Subsequently, the sample was put in toluene for three days, and the amount of absorbed solvent was used for the Flory–Rehner equation to determine the crosslink density. The calculation was corrected for the amount of fillers, which was determined by thermogravimetric analysis (TGA). Besides, the influence of fillers on the amount of absorbed toluene was compensated by the Porter correction [12]. (The Porter correction was chosen instead of the well-known Kraus correction [13] because of two reasons: firstly, that the Kraus correction is very dependent on an accurate value of the parameter ‘m’, which is related to the specific surface area of the carbon black in the rubber; secondly, Porter has shown that the correction as proposed by Kraus could be improved by the adjustments he made. For the Flory–Rehner parameter χ, the value 0.4 is chosen as a mean value for SBR, NR and BR with toluene. The density of the polymer blend is chosen as 0.95 kg/m^3^ as the mean value of the densities of these polymers according to the literature [14].

Verbruggen [6] investigated the applicability of this method for filled elastomer systems and indicated that it is accurate enough for engineering purposes, despite the fact that not all theoretical preconditions, like non-filled simple polymers, are met. For this analytical method, the average standard deviation was 0.025 for both the decrease in crosslink density and the sol fraction. The trends shown have been observed in many more samples.

#### 2.1.2. Evaluation of the Visible Particle Size

One of the quality-determining properties of the devulcanizate is the presence and size of particle cores, the so-called visible particles. For the estimation of the amount of visible particles and the size, the devulcanized material was blended with a styrene-butadiene/butadiene rubber (SBR/BR), carbon black-based tread compound on a 50/50 %wt base. This material was then sheeted out, vulcanized and visually rated according to the amount and size of visible particles. As reference, a 100% virgin compound was used, and the vulcanized samples were sorted according to their visible surface roughness. The observed differences were organized into 10 classes of increasing roughness, with Class 0 representing the virgin compound. Because of the low heat transfer-rate of rubber and the low distribution rate of TDAE, and hence, the DPDS, in the rubber particles, the work hypothesis was that the visible particles consist mainly of non-devulcanized material.

### 2.2. Process Development

#### 2.2.1. Influence of Devulcanization Time

**Figure 2:**
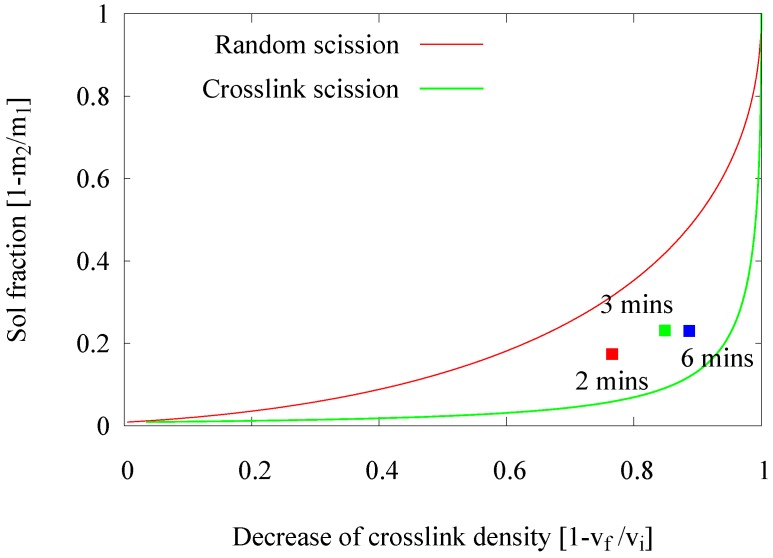
Influence of the residence time.

In an extruder, the devulcanization kinetics might be different due to differences in mechanical shearing forces, as well as heat transfer compared to an internal mixer. Therefore, an approach to re-adjust the devulcanization time was elaborated. In the earlier experiments in an internal mixer [1], the devulcanization time was optimized to 6 min. In this series of experiments, the residence time of the rubber in the extruder was varied by changing the extruder screw speed from 30–20 to 10 rpm, resulting in residence times of 2, 3 and 6 min. These screw speeds were chosen under the assumption that significant variations in residence time could be achieved without major variations in the shearing forces. An analysis of the devulcanizates was done by means of the Horikx plots, as shown in Figure 2. In this figure, only the results of one set of experiments are shown for clarity, which illustrates the trend found in many more series of experiments.

A shorter residence time results in a decrease of the devulcanization efficiency, indicated by the lower degree in ‘decrease of crosslink density’ due to the shorter devulcanization process. The position of the 6-min point in the Horikx plot indicates the highest degree in devulcanization and the lowest degree of main chain scission.

During the residence period, the dispersion of the devulcanization aid into the rubber, the transfer of heat into the rubber particles and the reaction of the devulcanization aids with the active chain fragments have to take place: after the scission of a crosslink, the devulcanization aid will react with the remaining highly-reactive radicals and neutralize these [15]. Only if all of these steps can be completed in the extruder, the devulcanization is complete, and a homogeneous devulcanizate can be achieved. A residence time of 6 min turned out to be the best choice in the extruder, as observed in the internal mixer, as well.

#### 2.2.2. Influence of Devulcanization Temperature

**Figure 3:**
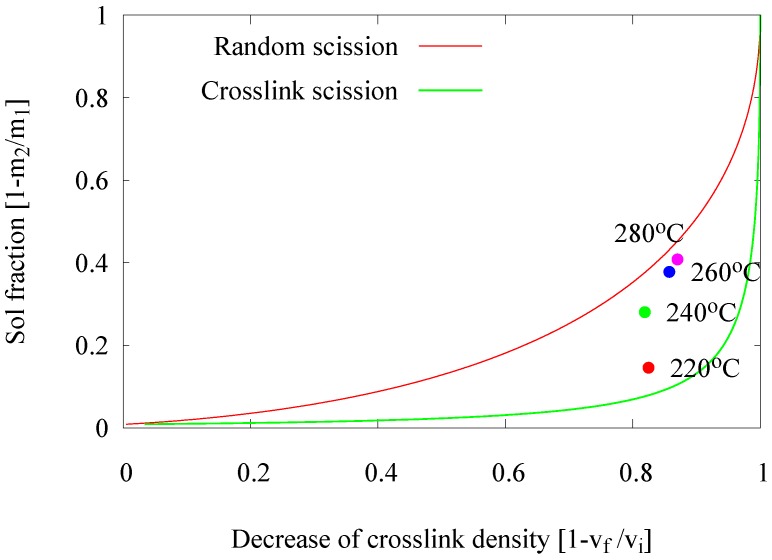
Influence of the de-vulcanization temperature.

Basically, in a thermo-chemical devulcanization process, there are three main factors contributing to the efficiency of the devulcanization: thermal energy, the chemical reaction and shearing forces. Initially, a devulcanization temperature of 220 ${\;}^{\circ }C$ was used, which is the optimal temperature found for the devulcanization in the internal mixer. The temperature in the extruder was set from 220–280 ${\;}^{\circ }C$ with the intention to optimize the devulcanization temperature with respect to the reaction rate. Therefore, the residence time for these experiments was set to 2 min. The influence of the process temperature on the devulcanization efficiency of GTR is shown in Figure 3.

The expected trend, that an increasing temperature improves the devulcanization efficiency, indicated by an increasing ‘decrease of crosslink density’, is not found. On the contrary, the data points show a tendency towards the ‘random scission line’, indicating that the quality of the devulcanizate decreases: at 280 ${\;}^{\circ }C$ nearly only main chain scission occurs.

In a thermo-chemical devulcanization process, the devulcanization agents are added in order to scavenge radicals formed during this process. However, at very high devulcanization temperatures, i.e., above 220 ${\;}^{\circ }C$, a more intensive generation of reactive radicals occurs. This threshold temperature indicates the optimum devulcanization temperature at which the rubber network can be broken in a controlled manner.

Therefore, it is to be concluded that the devulcanization temperature of 220 ${\;}^{\circ }C$ is the most efficient temperature for devulcanization in an extruder, as it was for the internal mixer.

#### 2.2.3. Influence of the Devulcanization Aid Concentration

**Figure 4:**
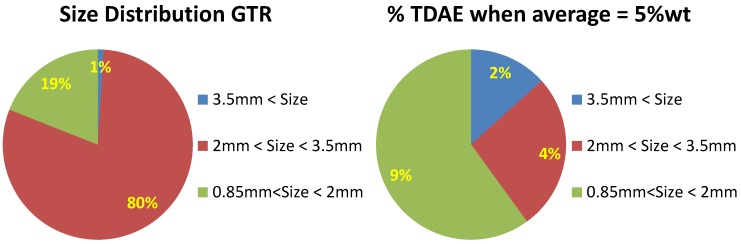
Size distribution of the GRT and the distribution of TDAE over fractions.

Compared to the material used in the earlier experiments [1] with a small and narrow particle size distribution, the GTR for these experiments had relatively large particles with a size distribution of 1–3.5 mm. During mixing of the GTR with the devulcanization aid dissolved in the TDAE, these will first be distributed over the surface area of the GTR particles before diffusing into the particles. As the larger particles have a much lower surface to volume ratio than smaller particles, it can be expected that the final concentrations of the devulcanization aid in the larger particles are much lower than in the smallest ones, because the diffusion rate of this viscous liquid into the GTR is very low. The size distribution of the GTR was measured by manual sieving: the major fraction of particles with a diameter of 2–3.5 mm was about 80 %wt of the sample, and the fraction with a diameter of 0.85–2 mm about 20 %wt. A small fraction of 1 %wt larger than 3.5 mm was found with this method (Figure 4 (left)).

**Figure 5:**
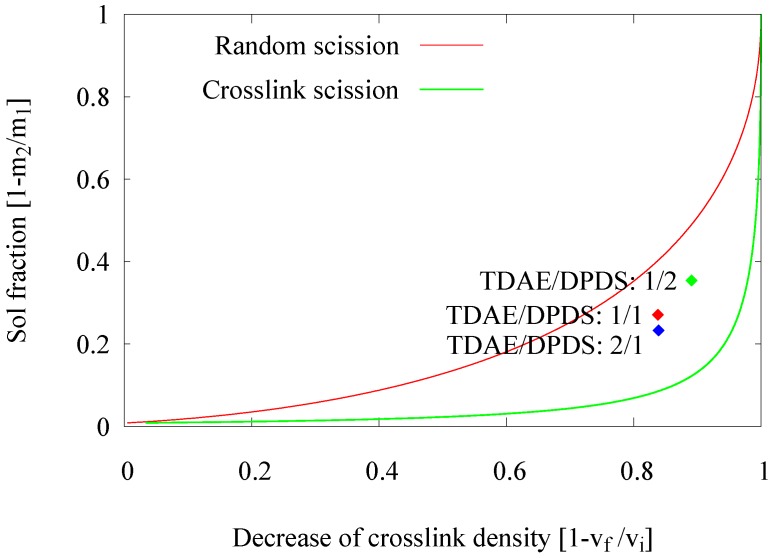
Influence of the concentrations of de-vulcanization aid and processing oil.

With the surface/volume ratio of particles with a diameter 3.5 mm set to one, the ratio for particles of 2 mm is 1.75. Hence, when distributing the devulcanization aid over the particles, 2 mm particles will have 1.75 times the amount compared to the larger ones, absorbing a disproportional higher amount of devulcanization aid compared to the relatively small fraction they constitute. It can be calculated and has been experimentally verified that for the main fraction of particles with a diameter of 2–3.5 mm to adsorb a TDAE concentration of 5 %wt, the amount of TDAE calculated for an average concentration of 5 %wt over the whole batch (Figure 4 (right)) should be increased to 6.2 %wt; hence, with a factor of 1.2. As the DPDS is dissolved in the TDAE prior to the mixing with the GTR, the same ratio is assumed. Hence, most experiments are performed with 18 ppm DPDS and 6.2 %wt TDAE instead of 15 ppm DPDS and 5 %wt TDAE. To account for this effect of particle size and the presence of still visible particles in the devulcanizate, experiments with an increased amount of DPDS and TDAE were performed. Increasing the amount of TDAE might improve the diffusion of the DPDS into the rubber matrix. The results are shown in Figure 5.

The data point in the Horikx plot representing an increased amount of DPDS has slightly moved to the right-hand side and closer to the main chain scission line. However, the change in the decrease of crosslink density lies in the error range of the analysis and is therefore not conclusive. Hence, the increasing amount of DPDS has a decreasing effect on the quality of the devulcanizate, while the amount of devulcanization remains more or less unchanged. Using a higher amount of TDAE oil was expected to improve the diffusion of DPDS in the rubber matrix resulting in a more homogeneous devulcanization. However, no difference in the effectivity or quality of the devulcanization were found; there is no significant difference between both positions. A plausible explanation can be that the shearing forces are less when the material has a lower viscosity due to the lubrication by the added oil. It can be concluded that a higher amount of devulcanization aid results in a higher ratio of crosslink to polymer break down. Additionally, a small decrease in the size of remaining visible particles was found.

This leads to the conclusion that increasing the concentration of DPDS from 18–30 mmol/100 g GTR with 6.2 %wt TDAE had for this material only a minor effect on the devulcanization efficiency and a negative effect on the quality. Hence, the concentration of DPDS of 18 mmol/100 g GTR with 6.2 %wt TDAE seems to be the best devulcanization mix.

### 2.3. Extruder Design and Parameter Optimization

#### 2.3.1. The Influence of Screw Speed

**Figure 6:**
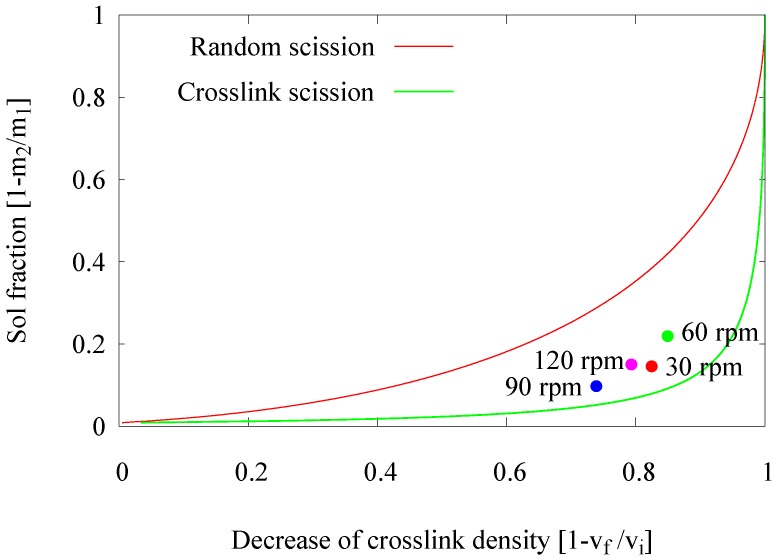
Influence of the screw speed.

In an extruder, an increase of the screw speed can affect several process parameters. These include local temperature, shear force and residence time. In a previous set of experiments to study the influence of the residence time, the screw speed was varied in only a very narrow range; assuming that the influence on the other, above-mentioned parameters is negligible. In this part of the experimental work, the intention was to study the actual effect of the screw speed, and the variation of the related process parameters was accepted. In this series of experiments, the screw speed was varied within a wide range of 30, 60, 90 and 120 rpm. The effects of screw speed and related parameters on the devulcanization efficiency of the GTR are shown in Figure 6.

An increase of the screw speed from 30–60 rpm causes the experimental data point in the Horikx plot to move upwards, representing an additional generation of the soluble fraction without significant reduction of the crosslink density. This might be due to significantly higher shear forces at 60 rpm, leading to uncontrolled break down of the polymer. The residence time was reduced by a factor of two; therefore, it was not expected and actually not found that the degree of devulcanization would be higher. However, when increasing the screw speed to 90 rpm and 120 rpm, the experimental data points in the Horikx plot moved backwards, indicating inefficient devulcanization.

These high screw speeds, thus low residence times of 60 s and 30 s, respectively, resulted in low degrees of devulcanization, as well as less polymer detached from the network. This is in contradiction with the expectation that high screw speeds create excessive shear forces and that therefore the soluble fraction should increase. However, the shear force is only one of the main parameters. The low residence times at high screw speeds might result in an inhomogeneous devulcanization due to a different devulcanization mechanism: peeling off the outer layers of the particles, while the inner cores of the particles stay more or less untreated at the initial crosslink density. In actual practice, this inhomogeneity causes a lower average decrease in crosslink density at a particular sol fraction than would have been obtained for homogeneous break down.

The final conclusion from this part is that the screw speed influences the devulcanization efficiency by the change in residence time more than by the variation of shear forces. Again the conclusion seems reconfirmed that a residence time of 6 min is necessary for completing the devulcanization reaction, resulting in rather low screw speeds for this type of extruder.

#### 2.3.2. Influence of the Screw Configuration

**Figure 7:**
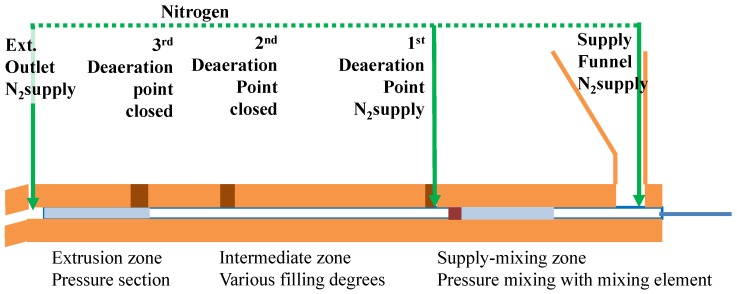
Low shear screw design and the positions of the de-aeration openings.

**Figure 8:**
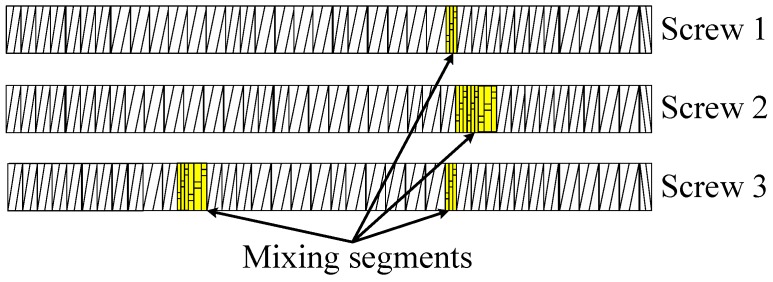
Overview of the three low shear screw designs.

The initial extruder experiments by Saiwari [1] were done with a screw design, which was originally optimized for ethylene-propylene-diene rubber (EPDM) devulcanization [16]. With this screw, a low devulcanization quality was found for the tire material; most probably due to the severity of the screw. In retrospect, this screw configuration may not have been the best choice, as GTR is very tough in comparison with the soft EPDM. Therefore, a logical consequence was to develop a less severe screw for GTR, to achieve a similar level of energy input from shearing. In this series of experiments, the screw configuration was newly designed and assembled as shown in Figure 7 and Screw 1 in Figure 8.

From the point of view that the processing in the extruder can be divided into three areas (Figure 7), supply and mixing zone, intermediate zone and extrusion zone, two alternative screw designs were tested, using the hypothesis that the still visible particles mainly consist of non-devulcanized material:

- Screw 2 with additional mixing elements in the supply and mixing zone, with the intention to improve the distribution of the devulcanization aids into the GTR particles.
- Screw 3 with mixing elements added to the intermediate zone, following the observations of high shear during the milling process reducing the still visible particles in size and with the intention to have the inner, non-devulcanized parts of the GTR particles exposed to the temperatures necessary for devulcanization during a maximum residence period. This implies that the inner temperature of the larger particles might have been too low in screw Setups 1 and 2 for an effective devulcanization due to the low thermal conductivity of rubber.

**Figure 9:**
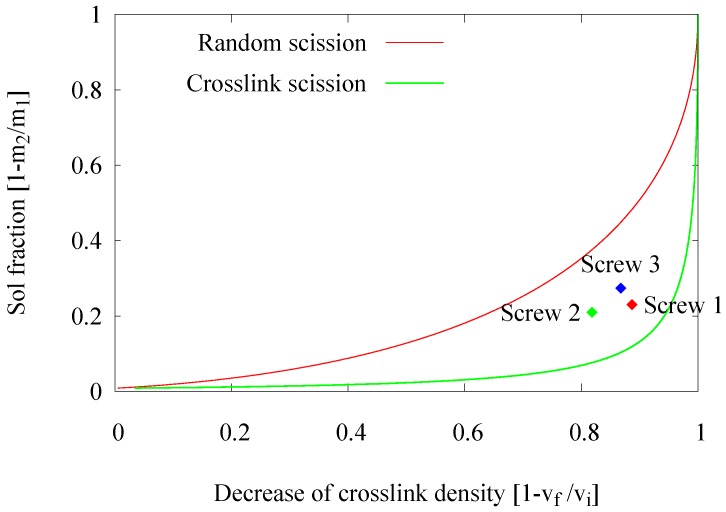
Influence of the screw configuration.

The results, for a devulcanization aid concentration of 18 mmol DPDS/100 g, are shown in Figure 9. Changing the screw configuration appears to influence both the devulcanization rate and quality and the amount and size of the still visible particles.

**Figure 10:**
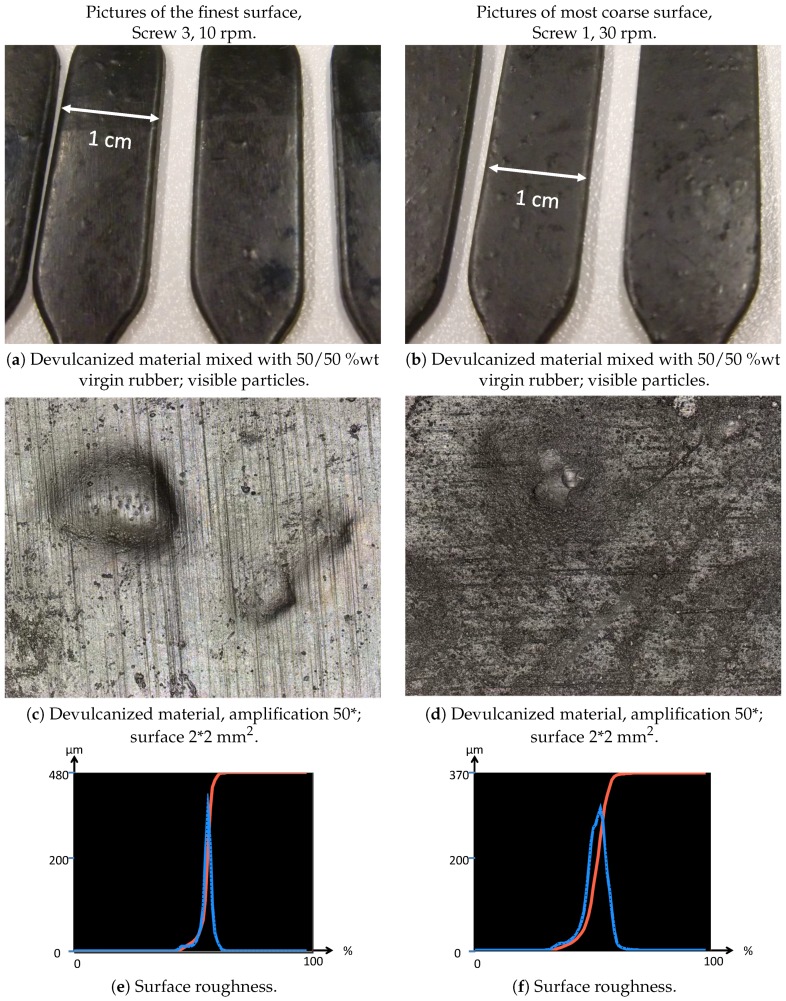
Plain (**a**,**b**) and laser scanning microscope pictures (**c**,**d**) of the finest and most coarse surfaces and the surface roughness distribution over the measurement surfaces (**e**,**f**).

**Figure 11:**
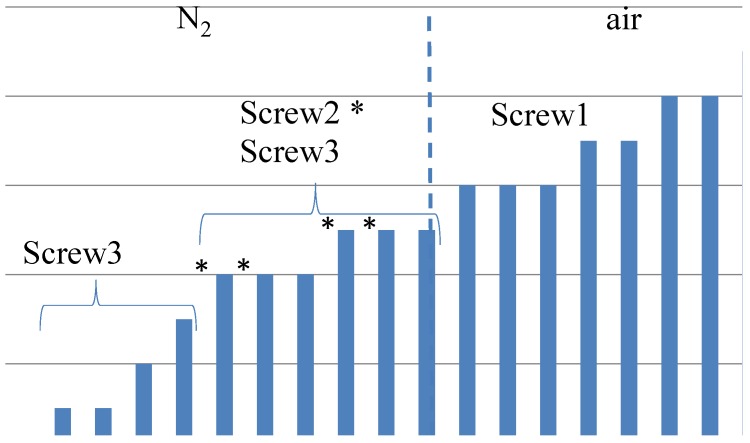
Relative order of the size and amount of the still visible particles by visual inspection. Scale indication: 0 = virgin rubber, 1 = best material so far (see Figure 10a), 10 = most and largest visible particles (see Figure 10b). * Material produced with Screw 2.

- Devulcanization rate and quality: According to the Horikx graph, the screw with Configuration 1 shows the best results. The screw with Configuration 3 shows a slight decrease in the quality of the devulcanizate compared to Screw 1, indicated by the increasing amount of sol for a comparable amount of decrease in crosslink density. For the screw Configuration 2, both the quality and decrease in crosslink density are inferior: the results in the Horikx plot show a decrease in devulcanization quality, as indicated by a relative decrease in distance to the random scission line.
- Visible particles: The investigation of the particle size of the devulcanizates was done and evaluated by visual inspection, both with and without a microscope. Without instruments, it can clearly be observed that there are large differences in the amount and size of visible particles on the surface of the re-vulcanized rubber blends; see Figure 10. The pictures in Figure 10 show the most extreme cases: the most homogeneous blend of devulcanized and virgin rubber (Figure 10a) and the material with the coarsest surface morphology (Figure 10b). To further quantify the surface morphology of the materials, laser scanning microscope pictures were used. Figure 10c,d shows a typical surface morphology of a 2 × 2 mm^2^ area: they visualize the quantity and size differences of the particles for the samples. Figure 10e,f shows the quantitative evaluation of roughness of the surface: the more homogeneous sample has only a few, though still large particles with a height of 200 micrometer on the surface; the distribution is rather narrow. The sample with the coarse surface has a broader distribution and a higher number of particles.
  A count of the visible particles particles as given in Table 2 shows that there is a significant difference in the number of large, as well as small particles. The total number of particles increases by approximately a factor of four from Category 1–10. For the further comparison of the sample morphology, the interval between Categories 1 and 10 is separated into nine steps of increasing size and the number of visible particles. A clear relationship between screw configuration and the quality of the surface could be observed based on these characteristic numbers: with Screw 1, the particles were the largest and most numerous, as can be seen in Figure 10b and Figure 11; with Screw 3, the best material was produced with the smallest and lowest number of visible particles, as is shown in Figure 10a and Figure 11. Class 0 was assigned to a quality comparable to that of virgin material. The results for Screw 2 were in-between those of Screw 1 and Screw 3; see Figure 11.

The results of Screw 2 indicated that the expected positive effect of the improved distribution of the devulcanization aid is hardly observable: there was only a minor decrease in the amount and size of the visible particles, and the quality of the devulcanizate decreased, as well. The results of Screw 3 showed that a small increase of shear in the devulcanization zone of the screw did decrease the quality of the devulcanizate as determined by the Horikx plot, but much less than expected. The effect on the amount and size of visible particles was as expected.

The results of the Screw 3 configuration indicated that a major part of the visible particles mainly consisted of non-devulcanized material. However, additional TGA and FTIR analysis of these particles showed a high silica content. This is the subject of further investigations.

Overall, it can be concluded that Screw 1 shows the best Horikx representation, but it also has the highest amount and largest size of still visible particles. Therefore, Screw 3, a low-shear screw with additional mixing elements in the devulcanization part of the extruder (see Figure 7 and Figure 8) turns out to be the most suitable screw configuration from the screw variations in this study for the devulcanization of GTR.

## 3. Experimental Section

### 3.1. Materials

The ground tire rubber (GTR) used in this investigation was obtained from Genan, Dorsten, Germany. It is a commercial passenger car tire grade, thus mainly based on synthetic rubber, and had a particle size of 1.0–3.5 mm. This GTR type was chosen due to its purity: a higher particle size material contained a considerable amount of fibers; a lower particle size granulate contained fine metal dust worn off of the grinding equipment and fine-ground stones, glass, etc.

Ambiently-ground car tire powder with a maximum particle size of 0.35 mm was used as a comparative material for a study on the effect of the visible particle cores. It was devulcanized and re-vulcanized similar to the coarser GTR. As this material behaved differently in the extruder compared to the 1.0–3.5 mm material, these results cannot be compared to the results of the granulate, other than the roughness of the re-vulcanized samples.

As devulcanization agent diphenyl disulfide (DPDS) was used and as the anti-oxidant, tris(2,4-di-tert-butylphenyl) phosphite (TDTBP). Both were supplied by Sigma Aldrich Cooperation, Zwijndrecht, The Netherlands. The processing oil treated distillate aromatic extract (TDAE) was supplied by Hansen & Rosenthal, Hamburg, Germany.

The solvents used for the analysis were acetone (purity >99.5 %wt), tetrahydrofuran (THF) (purity >99.8 %wt) and toluene (purity >99.8 %wt), all supplied by Atlas & Assink Chemie b.v., Enschede, The Netherlands.

### 3.2. Preparation of Devulcanizates

#### 3.2.1. Pre-Treatment

Prior to mixing with GTR, the TDAE, DPDS and TDTBP were molten and mixed at 70 ${\;}^{\circ }C$. Next, this blend was added to the cold (20 ${\;}^{\circ }C$) GTR. This was swollen and heated to 65 ${\;}^{\circ }C$ for 0.5 h or 24 h (depending on the experiment) in a Webac 2-roller-type heated sand mixer (Webac Gesellschaft für Maschinenbau mbH, Euskirchen, Germany).

#### 3.2.2. Extruder Setup

The devulcanization was performed in a KrausMaffei ZE 25 UTX co-rotating twin screw extruder (KrausMaffei Technologies GmbH, München, Germany), length 40D with D = 25 mm, facilitating 3 de-aeration positions between the supply funnel and outlet. The screw design was based on a minimal shear concept, as shown in Figure 7 and Figure 8, Screw 1.

When checking the progress of the devulcanization in the extruder with Screw 1 via the de-aeration openings in the barrel of the extruder, it appeared that the GTR kept its granulated shape up to halfway the extruder, after which the consistency changed into a smaller size granulate embedded in a plastified matrix at the end of the middle section of the extruder. Based on the observation in the internal mixer experiments that the devulcanization activity is decreasing considerably at temperatures below 180 ${\;}^{\circ }C$, the configuration of the extruder screw can be seen as consisting of three consecutive major sections: supply and mixing zone, intermediate zone and pressure or extrusion zone. In the supply and mixing zone, the temperature is kept at 180 ${\;}^{\circ }C$, in order to improve the diffusion of TDAE and DPDS while decreasing the chance of random polymer scission due to local high concentrations of DPDS. The intermediate zone is characterized by low shear forces due to the design of the screw, and the pressure or extrusion zone is needed for building up the extrusion pressure, hence by definition high shear.

In the supply and mixing zone, a mixing element was installed with the necessary preceding pressure elements. At the discharge side of the screw, pressure elements were added to be able to discharge the devulcanized product. In the intermediate section, a mix of several kinds of transport and pressure elements was used to create gentle mixing and shear due to varying degrees of filling of the screw in this zone (see Figure 7, a schematic of the extruder with the basic screw design, the de-aeration openings and the nitrogen supplies).

It is important to notice that in both the supply and mixing zone and the intermediate zone, the GTR is still present as particles. The behavior of these GTR particles in the extruder differs from the models concerning transport and mixing for high viscous liquids, and these models are therefore not applicable for the design of alternative screw configurations [17,18,19]. Depending on the degree of devulcanization, the GTR can still be in the particle shape in the extrusion zone.

#### 3.2.3. Additional Screw Designs

After the mixing step as described above, the material was milled on a two-roll mill with a nip-width down to 0.5 mm. During milling with this nip-width, it was observed that the visible particle size decreased considerably. However, the material did not become completely smooth; this indicated that, although the material is devulcanized, it preserved a granular structure up to rather high shearing forces. Because of the amount and size of the visible particles, additional screws were designed (see Figure 8): Screw 2 with additional mixing elements in the supply and mixing zone to promote the diffusion of the TDAE and DPDS into the granulate particles. Based on the observation of the decrease in the visible particle size in the milling step, Screw 3 was designed with additional mixing elements in the intermediate zone.

#### 3.2.4. Experimental Conditions

The temperature of the devulcanized material was measured at the outlet of the extruder with an integrated sensor in the extruder, which measured the temperature at the surface of the devulcanizate. For each different condition of the extruder, the temperature of the core of the devulcanizate was measured at the outlet of the extruder with an independent thermocouple-based thermometer. The core temperature was kept at the set temperature of the experiment $+2{\;}^{\circ }C\cdots +7{\;}^{\circ }C$. At higher screw speeds, the set temperature of the last section(s) of the extruder was adjusted to keep the core temperature within this range.

The order of the experiments was designed to first optimize the devulcanization temperature and subsequently the residence time, the concentration of the devulcanization aid and, at last, the screw configuration. For Screws 2 and 3, the residence times turned out to be similar to that of Screw 1 at the conditions used for the comparison of the screw performances.

#### 3.2.5. Post-Treatment

The die of the extruder is a rounded elongated slit of 20 mm × 40 mm. Through a pipe connection with a diameter of 70 mm and a length of 100 mm, the devulcanizate was either dumped into a cooling drum or directly put onto a cooling calender.

The possibility to quench the rubber after devulcanization, thus to rapidly cool it down to a temperature low enough to significantly reduce oxidation, proved to be one of the main factors contributing to a high devulcanizate quality in the batch process.

In the earlier work with an extruder, the devulcanizate was quenched into a water bath. Consequently, an extra drying step for the extruded devulcanizate was needed. This additional step at elevated temperature for a rather long period in the presence of oxygen resulted in low properties of the devulcanizate. Therefore, a cooling calender was designed and built to cool the devulcanizate directly after the discharge of the extruder.

The supply-funnel, extruder and extruder output piping were flushed with nitrogen during production.

#### 3.2.6. Surface Roughness

The devulcanizate was blended with a virgin rubber compound in a ratio of 50/50 %wt, based on polymer content, and vulcanized according to the recipe in Table 3 in order to compare the surface roughness, number and size of visible particles. Surface roughness and height measurements were done with a Keyence VK-0710 violet laser color 3D scanning microscope (Itasca, IL, USA). The surface roughness was determined according to ISO 4287 [20]. The amplification factor was 10, and the sample size was 2 × 2 mm^2^. Manual counting of visible particles was done on a surface area of 2 cm^2^.

## 4. Conclusions

The earlier established parameters of the devulcanization process in a small-scale, discontinuous batch mixer with a capacity of 15 g per batch were transferred to a 40D 25 mm co-rotating twin screw extruder process with a capacity of 2 kg/h. Within this scale-up of the devulcanization process, the following parameters were fine-tuned for the extruder-based process:

- Devulcanization process conditions: The extruder conditions turned out to be comparable to the parameters found for the Brabender internal mixer, as shown in Table 1:
  - Temperature profile: The main factor determining the devulcanizate quality is the temperature: if it is too low, devulcanization is too slow; if it is too high, the polymer degrades. The optimum within the temperature window investigated here was 220 ${\;}^{\circ }C$.
  - Time: The residence time in the extruder is another important influencing factor for the product quality: shorter times set by higher screw speeds result in insufficient devulcanization and uncontrolled breakdown of the polymer due to the higher shearing forces. The optimal condition for a screw configuration like Screw 3 is a 6-min residence time.
  - Devulcanization aid concentration: The optimum concentration of DPDS was found to be 18 mmol/100 g GTR. Increasing the concentration to 30 mmol/100 g GTR decreased the quality of the devulcanizate.

The conditions defined for the Brabender batch reactor were also valid for the extruder process. Looking at the degree of devulcanization, an extruder is more efficient than a batch mixer: while the maximum degree of devulcanization in the batch mixer was less than 80%, the percentages found after extruder devulcanization were between 80% and 90%. Higher degrees of devulcanization cannot be established, as some polymer is bound to the filler and is therefore not soluble; furthermore, the present devulcanization process is not efficient for monosulfidic crosslinks.

- Extruder parameters: The extruder parameters are interdependent: The screw configuration determines the amount of shear and the residence time, at a certain screw speed. For a small speed range, both residence time and shear rate can be assumed to have a linear relation with the speed; however, this is not valid for larger differences in screw speed.
  - Screw speed: For the given extruder and screw configuration and within a range of 10–120 rpm, 10 rpm leading to a residence time of 6 min was optimal.
  - Screw configuration: The low shear concept has been shown to be a useful approach. From the three screw variations tested, Screw 3, Figure 8, showed the best results in terms of devulcanization efficiency, quality and still visible particle size.
  - Fill factor: Being a paramount parameter for the amount of shear in an internal mixer, for the extruder screw, it appeared to be of less importance.
- Still visible particles: For non-optimal devulcanization parameters, large particles remain, which mostly consist of non-devulcanized material. However, even with the use of a small-sized passenger car tire powder, small visible particles remain in the devulcanized material. The first analysis indicates a high amount of silica in these particles, but additional investigations are necessary.

To be able to run the extruder under the best conditions for both the product and the environment, additional ancillary equipment is needed: swell equipment, nitrogen supply and a cooling calender.

## Figures and Tables

**Table 1:** Devulcanization conditions: Brabender internal mixer vs. twin-screw extruder. Values in bold are the standard values used in these investigations. Underlined values are the settings with the best results.

Factors	Optimized Brabender	Extruder Conditions
	Internal Mixer Conditions	
Devulcanization aid	DPDS, 15 mmol/100 g	DPDS 15, **18**–30 mmol/100 g
	compound	compound
Devulcanization oil	TDAE 5 %wt	TDAE 5, **6.2**–10 %wt
Anti-oxidant	TDTBP, 1 %wt	TDTBP **1 %wt**
Swelling time	30 min	**30** min and 24 h
Swelling temperature	65 °C	**65** °C
Devulcanization time	6 min	(<2), 2–**6** min
Rotor speed/screw speed	50 rpm	**10**–120 rpm
Devulcanization temperature	220 °C	**220**–280 °C
Devulcanization atmosphere	Nitrogen gas purging	In air
		nitrogen gas purging
Screw Configuration	High shear screw	Low shear screw with
	(first transfer of internal	additional kneading elements
	mixer conditions)	in devulcanization zone
Swelling equipment	Hot air oven	**Heated mixer**
Extrudate handling	Dumping in	Cooling in air
	liquid nitrogen	**Cooling in inert atmosphere**
		Cooling with cooling calender
Ventilation	-	Ventilation system with scrubber
		for cleaning and smell control

DPDS = diphenyl disulfide; TDAE = treated distillate aromatic extract; TDTBP = tris(2,4-di-tert-butylphenyl)phosphite.

**Table 2:** Amount and size of the visible particles for the finest and most coarse material.

Category (See Figure 11)	Material	Size	Amount
1	Finest:	≈1 mm	2.5/cm^2^
	(s3-10-12) *	>0.1 mm, <1 mm	5/cm^2^
10	Most coarse:	≈1 mm	8/cm^2^
	(s1-30-6)	>0.1 mm, <1 mm	22/cm^2^

* Sample coding: s3 = Screw 3 (1, 2 or 3), 10 = screw rpm (10, 20 or 30), 12 = indication of the devulcanization aid concentration (6 = base concentration, 9 ≈ 150%, 12 ≈ 200%; see Table 1).

**Table 3:** Blend for re-vulcanizing devulcanizate with virgin rubber. For virgin rubber only, the devulcanizate was not added, and the amounts of virgin rubber were doubled. Recipe according to ([1], Table 8.7).

Ingredients	Phr	Ingredients	Phr
Devulcanizate,	–	Zinc oxide	3.0
based on polymer content	50.0	Stearic acid	2.0
SBR	44.7	Sulfur	2.5
BR	17.5	TBBS	1.5
Carbon black N375	80.0	6PPD	1.0
TDAE oil	9.8	TMQ	2.0

TBBS = *N*-tert-butyl-2-benzothiazolesulfenamide; 6PPD = *N*-(1,3-dimethylbutyl)-*N*’-phenyl-p-phenylenediamine; TMQ = 2,2,4-trimethyl-1,2-dihydroquinoline.

## Abbreviations

The following abbreviations are used in this manuscript:

BR: Butadiene rubber
B.V.: Besloten Vennootschap, comparable with Ltd or Inc.
DPDS: Diphenyl disulfide , devulcanization aid
EPDM: Ethylene-propylene-diene rubber
GTR: Granulated car tire rubber
NR: Natural rubber
NWO: Nederlandse Organisatie voor Wetenschappelijk Onderzoek, The Netherlands Organization for Scientific Research
SBR: Styrene-butadiene rubber
TBBS: *N*-tert-butyl-2-benzothiazolesulfenamide, curative
TDAE: Treated distillate aromatic extract, processing oil
TDTBP: Tris(2,4-di-tert-butylphenyl)phosphite, antioxidant
THF: Tetrahydrofuran, solvent
TMQ: 2,2,4-trimethyl-1,2-dihydroquinoline, antioxidant
6PPD: *N*-(1,3-dimethylbutyl)-*N*’-phenyl-p-phenylenediamine, antiozonant
